# The relationship between physical activity and the severity of menopausal symptoms: a cross-sectional study

**DOI:** 10.1186/s12905-023-02347-7

**Published:** 2023-04-28

**Authors:** SongWen Wu, Yi Shi, Qiao Zhao, Ke Men

**Affiliations:** 1 Department of Public Health, Xi’an Medical College, 1st Weiyang Rd, Xi’an, Shaanxi Province 710021 China; 2 Shaanxi Provincial Centre for Disease Control and Prevention, Xi’an, 710054 China; 3 Department of Gynecology and Obstetrics, Xi’an Medical College, Xi’an, 710021 China

**Keywords:** Menopause, Symptom Assessment, Exercise, Random Forest, Women, Cross-Sectional Study, China

## Abstract

**Objective:**

To investigate the relationship between physical activity and the severity of menopausal symptoms in middle-aged women in northwest China.

**Methods:**

This was a cross-sectional online survey study. Using a snowball sampling method, 468 women aged 45 to 60 were recruited from northwest China and their demographic information was collected. The modified Kupperman Menopausal Index scale and International Physical Activity Questionnaire short form were used in this study. Random forest was used to rank the importance of variables and select the optimal combination. The direction and relative risk (odds ratio value) of selected variables were further explained with an ordinal logistic regression model.

**Results:**

The prevalence of menopausal syndromes was 74.8% and more than one-half of the participants had moderate or severe symptoms (54.3%). The Mantel-Haenszel linear-by-linear chi-square test showed a strong and negative correlation between physical activity level and the severity of menopausal symptoms (*P* < 0.001). Random forest demonstrated that the physical activity level was the most significant variable associated with the severity of menopausal symptoms. Multiple random forest regressions showed that the out-of-bag error rate reaches the minimum when the top 4 variables (physical activity level, menopausal status, perceived health status, and parity) in the importance ranking form an optimal variable combination. Ordinal logistic regression analysis showed that a higher physical activity level and a satisfactory perceived health status might be protective factors for menopausal symptoms (odds ratio (OR) < 1, *P* < 0.001); whereas perimenopausal or postmenopausal status and 2 parities might be risk factors for menopausal symptoms (OR > 1, *P* < 0.001).

**Conclusions:**

There is a strong negative correlation between physical activity and the severity of menopausal symptoms. The results have a clinical implication that the menopausal symptoms may be improved by the moderate-to-high level physical activity in the lives of middle-aged women.

## Background

Menopause is a period of life when the ovaries are depleted of oocytes and the cyclical activities of gonadotrophins, peptides, and steroids are lost[1]. The mean age of menopause is 51.4 years, but varies between populations from 49 to 52 years [2]. There are approximately 1.02 billion women over 50 years old around the world in 2021[3]. Based on the results of China’s seventh population census in 2020, there are about 688 million women in China and 247 million of them are over 50 years old [4]. Menopause has a significant effect on a woman’s endocrine, cardiovascular, skeletal, immune, and genitourinary systems [1]. Studies show that endocrine changes and bothersome symptoms such as sleeping problems and hot flushes put women at a higher risk of psychological symptoms [5], and affect their health-related quality of life [6]. Because about half of the world’s population will experience menopause, it is important to seek ways to increase physical, psychological, and social health during this life phase [7]. Following the findings from the World Health Initiative [8], which indicated that women using hormone replacement therapy had an increased risk of invasive breast cancer, the focus has shifted to finding alternative therapies. Physical activity is one such alternative and has been demonstrated to have physical, psychological, and social health benefits for middle-aged women [9].

The prevalence of menopausal symptoms can vary in women from different cultural, genetic, and regional backgrounds [10–16]. Women spend a third of their lives in the perimenopausal and postmenopausal stages [17], so investigating the relationship between physical activity and the severity of menopausal symptoms may help in relieving their symptoms and improving their quality of life [18].

Previous research on menopause-related health problems has been mainly conducted in Western countries, with few studies in China[19], especially in northwest China. The northwest region, where our study was conducted, differs in its cultural and socioeconomic characteristics from the rest of China, and there may be variations in the occurrence and intensity of menopausal symptoms [19].

The purpose of this cross-sectional study was to examine the relationship between physical activity and menopausal symptoms in middle-aged women in northwest China using the Mantel-Haenszel linear-by-linear chi-square test, random forest (RF), and ordinal logistic regression.

## Methods

### Study design

This cross-sectional online survey study was conducted with the questionnaire star from April 2022 to May 2022 among women living in northwest China. To reduce personnel mobility as per COVID-19 management measures, we distributed questionnaires online using a snowball sampling approach. First, we posted advertisements that described the purpose of the study and the principle of voluntary participation on well-known Chinese social media platforms (Tencent QQ and WeChat). Then we identified index individuals (30 participants) and, along with collected information on them by the questionnaire star, asked them to refer other persons suitable for the study. These named individuals were then recruited into the study. This process might end there or continue for further stages. Sampling continued until data saturation. The online informed consent has been obtained from all participate before collecting data.

### Participants

The inclusion criteria were native and middle-aged women between 45 and 60 years, no history of hysterectomy or oophorectomy, and the ability to understand a questionnaire. Pregnancy or lactation, menopausal hormone therapy or oral contraceptive use within the preceding 6 months, self-reported undiagnosed vaginal bleeding, serious illness (heart, brain, or movement) and cancer (breast, ovary, or cervix) were the exclusion criteria. We collected data through the Questionnaire Star platform. A total of 503 questionnaires were completed. According to the guidelines for data processing [20], 35 participants were excluded from the analysis. Therefore, 468 women were eligible for this study.

### Data collection

We collected data through the Questionnaire Star platform. The questionnaire consisted of three parts. The first part included self-designed questions about demographic and clinical data. The second and third parts are two validated, international questionnaires: the modified Kupperman Menopausal Index (mKMI) and the International Physical Activity Questionnaire Short Form (IPAQ-SF).

#### Demographic data

Demographic and clinical data, including age, residence, body mass index (BMI), education, history of chronic disease, menopausal status, parity, personal monthly income, perceived health status, job type, marital status, and physical activity, were collected through the questionnaire. According to the definition of the Stages of Reproductive Aging Workshop, the menopausal statuses were as follows: premenopause is defined as having regular menstrual periods; perimenopause is characterized by a persistent 7 days or more difference in length of consecutive cycles or an interval of amenorrhea of 60 days or more; postmenopause is the period after 12 consecutive months of amenorrhea [21]. BMI (kg/m^2^) was calculated and classified according to criteria from China’s Ministry of Health Disease Control Department (underweight, BMI < 18.5 kg/m^2^; normal, 18.5 ≤ BMI < 24 kg/m^2^; overweight or obesity, BMI ≥ 24 kg/m^2^) [22].

#### Menopausal symptoms

We assessed menopausal symptoms using mKMI, which consists of 13 items. The original KMI had 11 items, but the modified version adds the urogenital symptoms of urinary infection and sexual complaints. The original 11 items included sweating or hot flushes, palpitation, vertigo, headache, paresthesia, formication, arthralgia or myalgia, sleep problems categorized as somatic symptoms, fatigue, nervousness or irritability, and melancholia categorized as psychological symptoms. A scale ranging from 0 to 3 points is used to describe the severity of the complaints. The weighting factors in the mKMI are like those used in the original KMI. Two weighting points are given for each urogenital symptom. The total score ranges from 0 to 63, calculated as the sum of all items multiplied by the weighting factors. Scores ranging from 0 to 6, 7 to 15, 16 to 30, and greater than 30 were used to rate the degree of severity as none, mild, moderate, and severe, respectively [23]. In this study, Cronbach’s alpha was 0.84.

#### Physical activity assessment

The physical activity level was assessed using the IPAQ-SF [20]. It is a self-reporting questionnaire that asks about 3 specific types of activity, namely, walking, moderate-intensity activities, and vigorous-intensity activities. It is intended for adults aged 15 to 69 years. The test-retest reliability was 0.88 and the criterion validity was 0.92 in Chinese perimenopausal women [24]. The minutes spent every week on each type of activity are computed separately by multiplying the duration and frequency of the activity. A continuous activity score is calculated by multiplying the selected metabolic equivalent (MET) value and weekly minutes of activity, therefore expressing physical activity as MET-minutes per week. Physical activity level was divided into low, moderate and high according to IPAQ-SF methodology [20]:

High physical activity level:

- Vigorous-intensity activity on at least three days and accumulating at least 1500MET-minutes/week OR.
- Seven or more days of any combination of walking, moderate-intensity or vigorous intensity activities achieving a minimum of at least 3000 MET-minutes/week.

Moderate physical activity level:

- Three or more days of vigorous activity of at least 20 min per day OR.
- Five or more days of moderate-intensity activity or walking of at least 30 min per day OR.
- Five or more days of any combination of walking, moderate-intensity or vigorous intensity activities achieving a minimum of at least 600 MET-min/week.

Low physical activity level:

This is the lowest level of physical activity. Those individuals who not meet criteria for moderate or high physical activity level are considered low/inactive.

### Statistical analysis

Continuous variables with a normal distribution were presented as the mean ± standard deviation. For three or more groups with continuous variables, one-way analysis of variance (ANOVA) was used for comparisons. Categorical variables in different groups were presented as frequency and proportions and compared using chi-square tests. Because the data for physical activity level and the severity of menopausal symptoms were ordinal, the Mantel-Haenszel linear-by-linear chi-square test and Pearson’s correlation analysis were performed to estimate the relationship between the physical activity level and the severity of menopausal symptoms.

Random forest (RF) is a machine learning method proposed by Breiman [25] in 2001, which greatly improves the accuracy of classification and regression. Modeling using many independent variables often lead to complex models and overfitting [26]. Moreover, as the number of independent variables increases, the accuracy of models may decrease due to the increased noise and errors in the input variables. Before modeling, it is thus critical to select the variables that significantly contribute to improving the estimation accuracy, increasing the interpretability of models, and reducing the running time of the models [26]. RF provides a metric for judging the contributions of feature variables and increasing model accuracy.

Factors with a *P* value less than 0.05 in univariate and bivariate analysis were included in the RF model. The mean decrease in accuracy was calculated and used to rank variables by their importance. Multiple RF regressions were established by gradually increasing the number of variables in descending order of importance. The variables of the model leading to the smallest OOB error were selected and analyzed by ordinal logistic regression with *P* values, OR, and 95% CIs. All analyses were done with R 4.1.3 for Windows (R Foundation for Statistical Computing, Vienna, Austria). All *P* values less than 0.05 (two-tailed) were considered statistically significant.

## Results

### Baseline characteristics

The mean age of participants was 49.5 ± 4.9 years and more than one-half (290, 62.0%) ranged from 45 to 49 years old. Their mean BMI was 21.7 ± 2.5 kg/m^2^ and most (355, 75.9%) had a normal BMI. 199 (42.5%) women were graduated from university or college, 134 (28.6%) from high school and 135 (28.8%) from junior high school or below. There were 271 participants (57.9%) with a monthly personal income less than RMB 5,000. Most women were from the urban area (327, 69.9%), were physical laborers (292, 62.4%), were married or cohabiting (423, 90.4%), and had 1 to 2 children (84.0%). The mKMI score ranged from 0 to 44, with a mean of 12.1 ± 7.4. Although the severity of menopausal symptoms has 4 categories, moderate and severe were combined because there were few participants in the severe category, so 164 (35.1%) were in the moderate or severe group. The 3 menopausal symptoms severity groups significantly differed in terms of residence, history of chronic disease, menopausal status, parity, perceived health status and marital status (*P* < 0.05 each), as detailed in Table 1.

**Table 1:** Demographic and clinical characteristics by the severity of menopausal symptoms

Characteristics		Total (%)	The severity of menopausal symptoms (n, %)			${x}^{2}$(*P*)
		(n = 468)	None (n = 118)	Mild(n = 186)	Moderate or severe(n = 164)	
Residence	Rural	141(30.1)	29(20.6)	47(33.3)	65(46.1)	10.854 (0.004)
	Urban	327(69.9)	89(27.2)	139(42.5)	99(30.3)	
History of chronic disease	No	338(72.2)	98(29.0)	139(41.1)	101(29.9)	16.981 (0.002)
	Yes	75(16.0)	12(16.0)	28(37.3)	35(46.7)	
	Not known	55(11.8)	8(14.5)	19(34.5)	28(50.9)	
Menopausal status	Premenopausal	236(50.4)	79(33.5)	107(45.3)	50(21.2)	44.061 (< 0.001)
	Perimenopausal	132(28.2)	21(15.9)	42(31.8)	69(52.3)	
	Postmenopausal	100(21.4)	18(18.0)	37(37.0)	45(45.0)	
Parity	0	32(6.8)	14(43.8)	10(31.3)	8(25.0)	23.496 (0.001)
	1	195(41.7)	64(32.8)	76(39.0)	55(28.2)	
	2	198(42.3)	33(16.7)	82(41.4)	83(41.9)	
	≥ 3	43(9.2)	7(16.3)	18(41.9)	18(41.9)	
Perceived health status	No satisfied	202(43.2)	33(16.3)	69(34.2)	100(49.5)	42.469 (< 0.001)
	Generally	182(38.9)	50(27.5)	88(48.4)	44(24.1)	
	Satisfied	84(17.9)	35(41.7)	29(34.5)	20(23.8)	
Marital status	Never married/ separated/ divorced/ widowed	45(9.6)	16(35.56)	11(24.44)	18(40.0)	10.977 (0.027)
	Married/cohabitating	423(90.4)	102(24.1)	175(41.4)	146(34.5)	

### Relationship between physical activity level and severity of menopausal symptoms

The physical activity level based on the severity of menopausal symptoms is presented in Table 2. The Mantel-Haenszel chi-square value was 189.833 (P < 0.001), and the Pearson correlation coefficient (r) between the physical activity level and the severity of menopausal symptoms was − 0.638 (P < 0.001), indicating a strong inverse correlation.

**Table 2:** Relationship between physical activity level and severity of menopausal symptoms

Physical activity level	Total (%) (n = 468)	The severity of menopausal symptoms (n, %)			Mantel-Haenszel ${x}^{2}$ value(*P*)	Pearson *r* (*P*)
		None (n = 118)	Mild (n = 186)	Moderate or severe(n = 164)		
Low	92 (19.7)	3(3.3)	28(30.4)	61(66.3)	189.833 (< 0.001)	-0.638 (< 0.001)
Moderate	243(51.9)	19(7.8)	121(49.8)	103(42.4)		
High	133(28.4)	96(72.2)	37(27.8)	0(0)		

### Relationship between physical activity level and prevalence of menopausal symptoms

The prevalence of menopausal symptoms based on physical activity level is summarized in Table 3. Most of the participants (74.8%) had menopausal syndromes. More than one-half of the participants had moderate or severe symptoms (54.3%). The 5 most frequently observed symptoms were fatigue, sleep problems, sweating or hot flushes, nervousness or irritability, and arthralgia or myalgia. When symptom experience within each level of physical activity was compared, sleep problems were found to be the most frequent symptom among low physical activity women, fatigue was the most frequent symptom among moderate physical activity women, and sweating or hot flushes was the most frequent among high physical activity women. The occurrence of each menopausal symptom significantly differed in terms of physical activity level (*P* < 0.001 each). By pairwise comparison, it was found that the incidence of symptoms in the high physical activity group was lower than that in the moderate and low physical activity groups (*P* < 0.017).

The number of menopausal symptoms based on physical activity level are shown in Table 4. There was a negative linear relationship between the level of physical activity and the number of symptoms (*F* = 347.45, *P* < 0.001).

**Table 3:** Prevalence of menopausal symptoms by physical activity level

Menopausal symptoms	Total (%) (n = 468)	Physical activity level (n, %)			${x}^{2}$(*P*)
		Low (n = 92)	Moderate (n = 234)	High (n = 133)	
Somatic					
sweating/hot flushes	298 (63.7)	72 (78.3)^a^	168 (69.1)^a^	58 (43.6)	34.748 (< 0.001)
palpitation	165 (35.3)	57 (62.0) ^ab^	100 (41.2) ^a^	8 (6.0)	82.254 (< 0.001)
vertigo	225 (48.1)	62 (67.4) ^a^	147 (60.5) ^a^	16 (12.0)	97.986 (< 0.001)
headache	271 (57.9)	74 (80.4) ^a^	167 (68.7) ^a^	30 (22.6)	99.007(< 0.001)
paresthesia	153 (32.7)	55 (59.8) ^ab^	90 (37.0) ^a^	8 (6.0)	75.784 (< 0.001)
formication	110 (23.5)	42 (45.7) ^ab^	60 (24.7) ^a^	8 (6.0)	47.916 (< 0.001)
arthralgia/myalgia	284 (60.7)	72 (78.3) ^a^	173 (71.2) ^a^	39 (29.3)	77.987 (< 0.001)
sleep problems	311 (66.5)	84 (91,3) ^ab^	188 (77.4) ^a^	39 (29.3)	120.717 (< 0.001)
Psychological					
fatigue	319 (68.2)	79 (85.9) ^a^	190 (78.2) ^a^	50 (37.6)	81.818 (< 0.001)
nervousness/irritability	298 (63.7)	83 (90.2) ^ab^	184 (75.7) ^a^	31 (23.3)	136.961 (< 0.001)
melancholia	204 (43.6)	64 (69.6) ^ab^	125 (51.4) ^a^	15 (11.3)	87.806 (< 0.001)
Urogenital					
urinary infection	105 (22.4)	46 (50.0) ^ab^	58 (23.9) ^a^	1 (0.8)	76.389 (< 0.001)
sexual complaints	165 (35.3)	46 (50.0) ^a^	101 (41.6) ^a^	18 (13.5)	40.490 (< 0.001)

^a^*P* < 0.017 high versus low and moderate; ^b^*P* < 0.017 moderate versus low.

**Table 4:** Number of menopausal symptoms by physical activity level

Physical activity level (n, %)	Numbers of menopausal symptoms	*F*	*P*
Low	9.09 ± 2.55	209.41	< 0.001
Moderate	7.21 ± 2.75^a^		
High	2.41 ± 1.84^ab^		

^a^*P*<0.05 low versus moderate and high; ^b^*P*<0.05 moderate versus high.

### Variable rank selection using RF

**Fig. 1:**
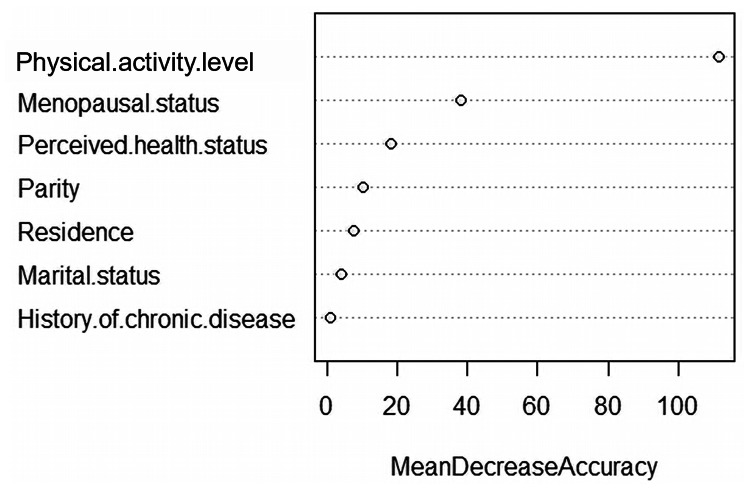
The importance ranking of feature variables

**Fig. 2:**
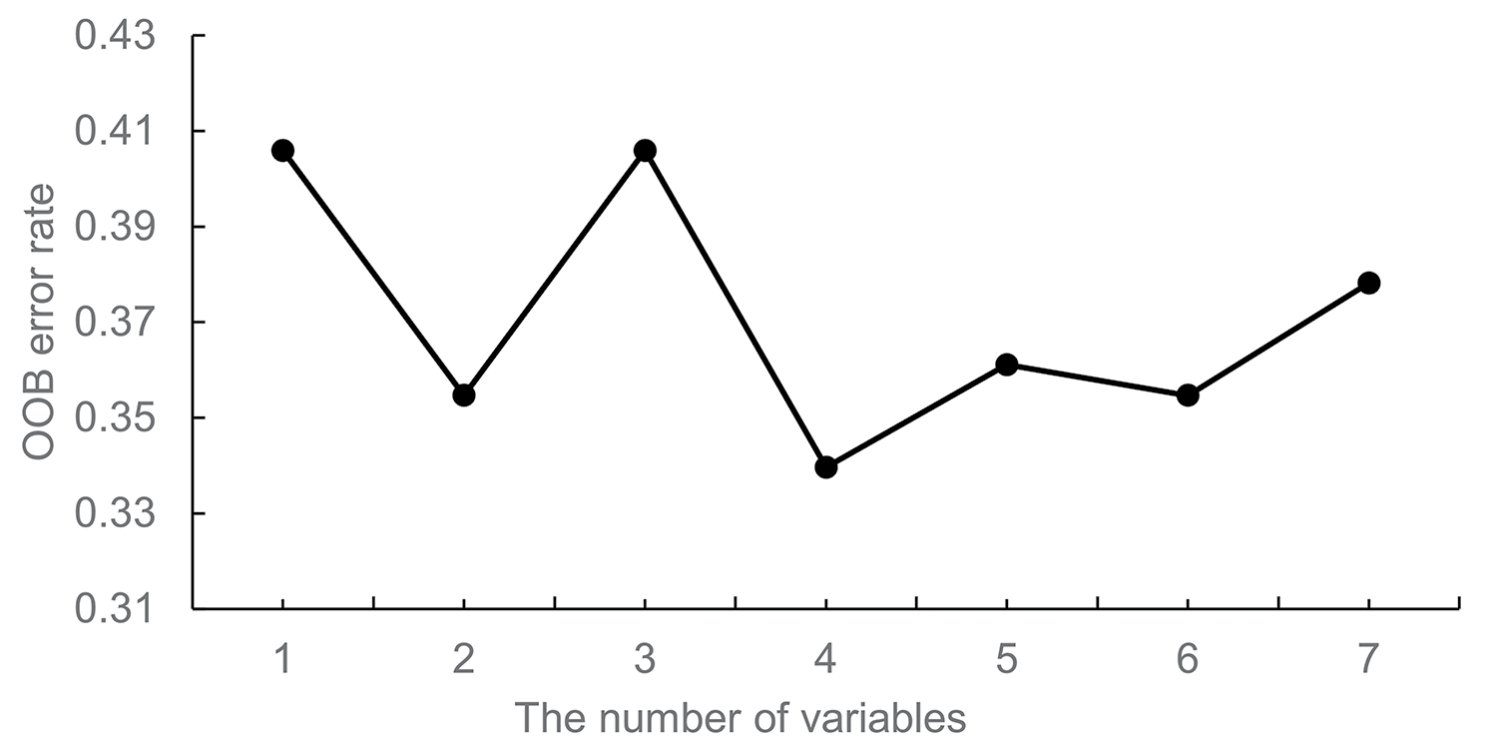
The RF regression results (OOB error rate) of feature variables

Seven feature variables with *P* < 0.05 in univariate analysis were included in the RF model. Figure 1 shows that among the 7 feature variables, RF regression identified the most significant feature variable associated with menopausal symptoms severity as physical activity level. Figure 2 shows that the OOB error rate reaches the minimum when the top 4 variables in the importance ranking form a variable combination.

### Factors associated with the severity of menopausal symptoms

The 4 most important variables, i.e., physical activity level, menopausal status, perceived health status, and parity, were included in the ordinal logistic regression model. Analyses were performed to investigate independent factors associated with the severity of menopausal symptoms. Table 5 shows that all 4 selected variables are significantly correlated with the severity of menopausal symptoms. The severity of menopausal symptoms decreased in participants with higher levels of physical activity (Moderate: Low OR, 0.26; 95% CI, 0.15–0.45; High: Low OR, 0.0083; 95% CI, 0.0038–0.017). Menopausal status was positively correlated with the severity of menopausal symptoms (Perimenopausal: Premenopausal OR, 2.65; 95% CI, 1.65–4.27; Postmenopausal: Premenopausal OR, 2.45; 95% CI, 1.48–4.10). A negative association was found between perceived health status and menopausal symptoms severity (General: Not satisfied OR, 0.61; 95% CI, 0.40–0.94; Satisfied: Not satisfied OR, 0.41; 95% CI, 0.23–0.74). Women with 2 children had an increased risk of more severe menopausal symptoms (OR, 2.32; 95% CI, 1.09–5.59). The accuracy of the ordinal logistic model was determined by the receiver operator characteristic (ROC) curve, which showed an area under the curve (AUC) of 0.83.

**Table 5:** Ordinal logistic regression analysis for factors associated with the severity of menopausal symptoms

Variables	*P* value	OR	95%CI
Physical activity level			
Low		1.00	
Moderate	< 0.001	0.26	0.15 to 0.45
High	< 0.001	0.0083	0.0038 to 0.017
Menopausal status			
Premenopausal		1.00	
Perimenopausal	< 0.001	2.65	1.65 to 4.27
Postmenopausal	< 0.001	2.45	1.48 to 4.10
Perceived health status			
No satisfied		1.00	
General	0.026	0.61	0.40 to 0.94
Satisfied	0.0031	0.41	0.23 to 0.74
Parity			
0		1.00	
1	0.67	1.20	0.52 to 2.86
2	0.028	2.32	1.09 to 5.59
≥ 3	0.18	2.02	0.72 to 5.79

## Discussion

In this cross-sectional online survey, we explored the relationship between physical activity and the severity of menopausal symptoms among women aged 45 to 60 years in northwest China. Both bivariate and multivariate analyses showed that a high physical activity level were associated with less severe menopausal symptoms. Our results are in line with previous studies [19, 27]. This association may be mediated by several mechanisms, including enhanced brain aminergic synaptic transmission, increased levels of endorphin, improved self-efficacy, diversion from stressful stimuli, microRNAs regulated physical activity-induced bone remodeling and improved mitochondrial function [28–30]. Many studies analyzed underline the importance and beneficial effects of physical activity on menopausal symptoms. However, a randomized controlled study with 6 months of moderate physical activity intervention showed that exercise is not an effective treatment for hot flushes and night sweats, but a definitive statement could not be made until more evidence was available [31]. A cross-sectional study found that the moderate activity group had the fewest menopausal symptoms, followed by the high activity group and the low activity group; thus, the relationship between physical activity and menopausal symptoms showed a U-shaped trend [32]. Evidence suggests that different types, intensities, and frequencies of physical activity might have different effects on menopausal symptoms, but few high-quality studies validated these observations [33]. Further studies to clarify these inconsistencies are needed.

Additionally, some baseline demographic and clinical characteristics, such as perimenopausal or postmenopausal status, lower satisfaction with perceived health status, and 2 parities, were also identified as risk factors for severe menopausal symptoms. Menstrual cycle disorders and menstrual volume changes affect women’s physical and mental health [1]. A study in China found that middle-aged women experience the first menstrual change earlier than their first menopausal symptoms by about 11.1 ± 9.2 months. If effective intervention is given during this menstrual change period, menopausal symptoms may be improved [34]. Our study also found that women with 2 children had the most severe menopausal symptoms, which was somewhat different from other studies [11]. This may be because when China implemented the 3-child policy, most of the participants in this study had passed the optimal childbearing age, so the number of people who had 3 or more children was only 7, which may have an impact on the research results. Further epidemiological surveys and fundamental research should be conducted to provide more evidence for clinical practice.

In the process of modeling, we used the two-step algorithm proposed by Genuer et al. to select variables with RF [35]. Firstly, we ranked the 7 variables by sorting them in descending order of importance (physical activity level, menopausal status, perceived health status, parity, residence, marital status, and history of chronic disease). We then computed the OOB error rates of RF for the nested models starting with the most important variable (physical activity level), and ending with the one involving all important variables kept previously [35]. The top 4 variables (physical activity level, menopausal status, perceived health status, parity) that led to the smallest OOB error were selected for the construction of the model and used for interpreting the results. RF does not overfit and has no restrictions and assumptions on the data. It can rank the importance of the factors with high prediction accuracy, but its interpretation is poor [36]. The ordinal logistic regression can make a more intuitive explanation of the influencing factors [36]. This study combines RF and ordinal logistic regression modelling of menopausal symptoms. It provides a new modelling approach for similar studies.

To the best of our knowledge, the current study is one of few studies which has investigated the association between physical activity level and severity of menopausal symptoms in middle-aged women in northwest China using validated instruments [19]. The most special feature of this study was that the importance of factors was ranked by the RF. But some limitations exist. First, this was a cross-sectional design that did not permit assessment of the temporal and potentially causal relation of variables. Second, the data were self-reported; however, we used the usual methods in the literature. Third, potential selection bias could exist because the participants who completed the survey were those who had internet and smartphone access. Fourth, we investigated this association in only one region in China, so the generalization of our findings to other areas and ethnicities should be made with caution.

## Conclusion

Our study showed that among middle-aged women in northwest China, a higher level of physical activity is associated with less severe menopausal symptoms. Based on RF ranking and screening, physical activity level was identified as the most important factor affecting the severity of menopausal symptoms. Our study provided useful information for physicians and policy makers on devising specific programs to encourage middle-aged women to do moderate to high levels of physical activity.

## Data Availability

All data generated or analyzed during this study are included in this published article.

## List of abbreviations

RF: Random forest
mKMI: Modified Kupperman Menopausal Index
IPAQ-SF: International Physical Activity Questionnaire short form
BMI: Body mass index
MET: Metabolic equivalent
ANOVA: Analysis of variance
OOB: Out-of-bag
